# Arthritis, or Gout

**Published:** 1838-03-14

**Authors:** N. Chapman

**Affiliations:** Professor of the Theory and Practice of Physic, in the University of Pennsylvania


### MEDICAL EXAMINER.

DEVOTED TO MEDICINE, SURGERY AND THE COLLATERAL SCIENCES.

**EDITED by J. B. BIDDLE, M. D. and M. CLYMER, M. D.**

No. 6. PHILADELPHIA, WEDNESDAY, MARCH 14, 1838. Vol. 1.

### ARTHRITIS, OR GOUT.

*By* N. Chapman, M. D., *Professor of the Theory and Practice of Physic, in the University of Pennsylvania.*

(Continued from page 76.)

Gout is sometimes so faintly or ambiguously exhibited in its regular or articular form, as not to be readily recognized. It may be mistaken for any phlogosis or irritation of a joint, and can only be discriminated by a careful perquisition into the case. Not a little may be learned from the precursory symptoms—sometimes from the existing appearances, and particularly from the affection being permanent or otherwise. Gout is nearly always changeable, while the irritative lesions, with the exception of the rheumatic, are the reverse, or fixed till their subsidence.

It is rheumatism, under all circumstances, which bears the closest affinity to it. But when we come to the history of that disease, we shall find they vary so materially, that they need not be confounded.

The leading particulars in which they differ, I shall only mention. Gout belongs chiefly to the higher class of society, is the result of a series of habits which vitiates the nutritive processes, or is received as an inheritance without sometimes any obvious excitements, and once occurring, is apt to be repeated throughout life at certain intervals. On the contrary, rheumatism is met with as exclusively among labouring people, and seems to have as an only cause, the influence of cold in some mode of application. The former is pretty uniformly preceded by derangement of health, and the latter not, and is wanting in the same liability to stated recurrences. The common seat of the one is in the smaller, and of the other in the larger joints, and though in each instance, swelling takes place with great suffering, these incidents are not identical; gouty inflammation being far more bright, florid, and polished, with a greater tendency to edema, and the pain is peculiarly gnawing and lacerating. The distinction, however, on which most reliance may be reposed, is, that gout always originates internally, and rheumatism very seldom. There is however a compound form of the disease exceedingly perplexing, induced by rheumatism falling on joints, previously weakened by arthritic attacks, causing what might be properly called rheumatic gout.

In its regular form, gout is rarely, or perhaps never immediately fatal. As I have said, its tendencies indeed are mostly salutary, clearing away other affections and re-establishing, for a time, the order of health. Death occurring from it, some important vital part becomes affected, or the constitution is gradually undermined, and some one of various affection, as its consequence, is induced, ending mortally.

Concerning the issue of an attack, therefore, it may be presumed, that it will be favourable, when the constitution is sound, particularly the viscera, and when the disease, entirely quitting the interior, becomes immoveably fastened on the extremities. Convalascence is denoted by the tongue becoming clean, with a return of appetite, the stools more natural, lateritious deposits in the urine, the skin relaxed, and with these, a subsidence of vascular and nervous irritations, and of the local intumescence and inflammation.

The adverse circumstances are, of course, pretty much the contrary,—as a decayed system, weakness of the primæ viæ, or an imperfect translation of the disease outwardly, or fluctuation in the seat of it, or proneness to recede from the joints to the internal organs, attended by irritative fever, and much nervous and mental inquietude.

The anatomical characters in gout might be inferred with some accuracy, from the preceding account. Death seldom taking place in the acute stage of it, except by a metastasis to vital organs, by which the joint is relieved, of course, we know little of the articular appearances. In the more recent attacks, however, inflammation with a peculiar albuminous sort of exudation, or serous effusions, has been noticed in the joints. Chronic cases present every variety of structural lesion. The synovial membrane is essentially altered, the bursæ mucosæ enlarged, and indurated, the cartilages absorbed, the exposed surfaces of bone smooth and polished, or where such destruction has not occurred, a firm anchylosis, wasting of the ligaments, or the reverse, thickening of them, and throughout the articulation, deposits of earthy or saline matter, generally the uvate of soda. As regards the internal lesions, it may be enough to state, that the stomach and bowels, the liver, lungs, heart and brain, are occasionally met with in various degrees of inflammation only, or of disorganization.

It is not necessary that I should enter, to any extent, into the controversy which has so long prevailed, relative to the pathology of this disease. Till the time of Cullen, who satisfactorily refuted the hypothesis, it was, with scarcely an exception, regarded as originating in some morbid condition of the blood, by which a humour, an acid, or an alkali, or some acrid salt was generated, and dropped into the affected part. The very term *gout*, as we have seen, expresses such a notion. These suggestions of a crude pathology are so completely set aside, that I shall dismiss them without further remark.

My own conviction is, that it is primarily an affection of the digestive organs, and intimately connected with Lithiasis, or the calculous complaints generally.

Both diseases are induced by a train of similar causes, and are prevented or palliated by similar means. It has also been shown, by the analyses of the ablest chemists, that the calculous concretions in the kidney or bladder, and those which form in the joints, called *chalkstone*, are, sometimes, identical, being mostly urates of soda. Not unlikely, the same diathesis is common to the two diseases, and the product of it may be thrown off by the kidneys, or the skin; in confirmation of which, we learn that the urine and perspiration in each case, have an excess of acid, the uric or phosphoric, most generally. These emunctories not adequately performing their offices in this respect, an accumulation of the acid results, and calculous formations take place in the kidneys, bladder, or articulations, or in each, as may happen. The latter, however, is a rare event.

Generally, the kidneys and skin are vicarious in their operations, and where the powers of the one fail, those of the other are correspondently invigorated. Let it not, however, be supposed, from the views here presented, that any countenance is lent to the notion formerly alluded to, of the dependence of the gouty paroxysm on such deposites in the joint. These must be deemed the effect, not the cause of the disease, which is shown to be true, independently of other consideration, by the fact, that they occur at the close, not at the commencement of an attack, and for the most part, are altogether wanting.

As previously intimated, gout has its origin in a vitiation of the digestive organs, and whatever subsequently arises, is to be traced up and assigned to this starting point, as the "*fons et origo mali.*" Thus the local affection of the joints, as well as the excitation and fulness of the vascular system, so frequently the concomitants on gout, as to be considered by some, as really constituting the disease itself, I hold to be merely secondary and subordinate. Of the precise nature of this primary lesion we are imperfectly informed. The advocates of the hypothesis have, with a single exception, offered no explanations of it. Broussais, to whom I now allude, in conformity with his dominant propensity to generalize, declares it to be an inflammation of the gastro-enteric mucous surface, to which indeed, he seems to impute all things. That such a condition sometimes ultimately takes place, we are not permitted to doubt, though it is equally true, that more frequently it is merely a functional derangement, ever analagous in its leading features to the genuine forms of dyspepsia. Brown indeed calls it, "the indigestion of the luxurious." It is hence very probable, that in common with the dyspeptic affection, the inception of gout is to be referred to the centre of the ganglionic nerves, and which disturbance exercises a very material influence over the case throughout its whole career. Looking at the causes of the disease, the phenomena of its rise and progress, and in some respects, the method of cure, the conclusion can scarcely be resisted.

On the extremities becoming affected, the internal irritation is mostly relieved, and as happens in the exanthemata, such is the natural process of cure. This opinion differs from that of some of the modern pathologists, who maintain, that the topical affection is primary, leading to constitutional derangement, or that, as is thought by others, it is merely a symptom, excited and kept up by the persistence of the irritation in the internal tissues. These hypotheses, partially applied, are correct. Denying the originality of the local lesion, it is yet not unreasonable to suppose, that it may operate to the aggravation of the general disturbance of the system, and it is more apparent, that there are cases of an occasional occurrence, where the disease, still lingering in a degree in its primary position, may have the effect imputed to it. Conceding this, my views remain uninvalidated as having reference only to those instances of the disease, where the metastasis is complete.

The seat of the local affection being among the parts directly subservient to the articulation of the joints, it has many of the characteristics of the inflammation of the fibrous textures. Whatever may be its violence, genuine arthritic action, strictly confined to these textures, never ends as common phlogosis, in the effusion of serum or lymph, or the secretion of pus. These taking place, the inflammation has invaded other tissues, the surrounding cellular, or the serous synovial membranes. Its proper terminations are such as previously noticed. Yet it must be confessed, that there are very striking singularities in gouty action, and that the whole subject is not without obscurity. Located in precisely the same parts as rheumatism, and various other inflammatory affections, it is so far from having an identity with them, that usually it may be at once discriminated. As an attempt merely at an explanation of these differences, I shall offer the conjecture, that the idiosyncracy in question is owing to the peculiarity of the causes of gout, it being determined that in the modification of morbid action, the cause of it has less influence, than the structure which it may occupy. Not satisfied, however, with this exegetical suggestion, it has been advanced, that the whole affection is purely a neuritis or neuralgia. That the nerves may be implicated in it, in common with all other phlogoses, I have shown to be probable, though to refer the entire case to such a state, were surely a very hasty conclusion from a very superficial examination, warranted neither by the prominent symptoms, the phenomena on dissection, nor the mode of cure. But more of this, when we come to rheumatism.

Consulting most of the writers on the subject, it will be perceived, that little is prescribed in the paroxysm of this disease, and that it has become too much the practice every where, to let it spontaneously expend itself. To envelop the limb in flannel, and to urge a patient endurance of the pain and confinement, constitute, indeed, proverbially, what is mostly done in podagra. That a system so inert, could only have arisen from the want of confidence in the known remedies, or to a conviction of the injurious effects of intermeddling at all with the case, is apparent. As long ago as the time of Ovid, and from the same causes, such an opinion seems to have been entertained. The poet at least declares:

"Tollere nodosam nescit medicina podagram."

Experience, however, has taught me, that much can be effected in the paroxysm, and that we are called upon to use remedies as unhesitatingly in it, as in any other acute inflammatory affection. To this state of the disease, I am, in the first place, to address my remarks.

Of all the means, which I have ever tried, the most decidedly efficacious is active purging. This is a very ancient practice, having prevailed with no interruption, from the earliest times, till it was prohibited by Sydenham, on purely theoretical grounds, it being, he observed, an invariable law of nature, that the peccant matter of the disease should be thrown out by the extremities, emetics and cathartics will have no other effect than that of counteracting this design.

Need I observe, that the example of Sydenham in this case, has been highly pernicious, having led to the desertion of a practice which, if judiciously applied, is safe, and particularly calculated to overcome this most distressing disease.

I have before stated, that gout is intimately connected with depraved conditions of the alimentary canal. Whether this opinion be correct or not, it may be confidently affirmed, that the practice it dictates is sound, and fully warranted.

For upwards of thirty years, I have habitually employed purgatives in the paroxysms of gout, and with unequivocal advantage. Not content with simply opening the bowels, I completely evacuate by purging the entire alimentary canal. This being accomplished, the distressing sensations of the stomach are usually removed; the pain and inflammation of the limb gradually subside, and the paroxysm, thus broken, speedily passes away. To effect these purposes, however, it is often necessary to recur to the remedy repeatedly. As to the particular purgative, this will depend on circumstances. Considerable depravation existing in the chylopoietic viscera, I prefer a dose of calomel, or the blue pill, to be hastened in its operation by magnesia, alone, or with Epsom salts. The mercurial purge is seldom again required, and the bowels may be kept freely open by any of the milder laxatives. Generally, however, the most effectual of this class of articles, and especially with a view to the immediate reduction of the topical phlogosis and pain, is the following mixture, a table spoonful of which should be given every hour, till it purges copiously.

| | | |
|---|---|---|
| ℞ | Magnes. Calc. | ʒi |
| | Sulph. Magnes. | ʒij |
| | Vin. Sem. Colch. | fʒss |
| | Aq. Cinnam. | f℥iij |
| | | f. sol. |

Eminently beneficial as an evacuant undoubtedly, I do not think, that all its effects are ascribable to this mode of operation. Colchicum has a peculiar property to which some of the credit must be assigned. Given alone, it is less serviceable, and, hence, we are to seek in a combined influence, for the salutary tendencies of the mixture.

My practice with regard to purging in gout, differing materially from the prevalent one, it may, perhaps, be expected, or at least desired, that I should justify it on other grounds than my own declaration of its efficacy. To its defence I can bring both reason and authority.

Granting the disease to be preceded or accompanied with those symptoms of depraved stomach and bowels, which I have noticed, no one can doubt for a single moment of the propriety of resorting to the use of purging. There is here, indeed, that group of affections, which the common observation of practitioners has taught is most readily removed by the thorough evacuation of the alimentary canal.

Why we should adopt a different course in gout, and refuse the remedies we have found so efficacious under nearly similar circumstances, in other diseases, is a question which, I suspect, cannot be very satisfactorily answered. It may be further urged, in support of the purgative plan, that, conformably to a very general observation, the paroxysm is always abated, if not removed, by the coming on of diarrhœa, or dysentery, or cholera morbus. This interesting fact did not escape the notice of Hippocrates, who tells us, that the disease is never cured, where the bowels are *constipated*, except by the providential occurrence of a dysentery, by which he seems to mean any spontaneous purging, and further states, that he had witnessed the best effects from the profuse discharges, which he emphatically calls a "melting down of the bowels." It is also recorded by Musgrove, one of the earliest and best of the professed writers on gout, that "during the paroxysm" a diarrhœa often takes place, which carries off the tumefaction and pain. "Cures effected in this way," he remarks, "have the advantage, that they do not return for a long time afterwards."

The confession, on this subject, of Sydenham himself, is exceedingly curious. Treating of gout, suspended by diarrhœa, he advises, as the only means of restoring the paroxysm, which he deems very essential to health, to give certain medicines to check the discharge from the bowels, which, he says, when completely stopped, the gout comes "thundering back on the extremities."

Nothing can be more conclusive, at least, of the efficacy of diarrhœa, in removing the arthritic paroxysm, than this acknowledgment.

It has already been said, that the treatment of gout by purgatives is not a new practice. My time, however, will not allow me at present, to offer to you, in detail, a retrospective history of what has been done in this way. It may, perhaps, be sufficient to state, what I have before done in some degree, that, commencing with Hippocrates, the practice continued till it was put down by the proscription of Sydenham.

During this long period, it was pursued successively, by the physicians of Greece, of Rome, of Arabia, and of modern Europe, after the revival of letters. The whole of them concur in the propriety of the practice, and some, in speaking of its success, use, if not the language of enthusiasm, certainly the tone of entire confidence.

Nor is it unworthy of recollection, that the purging in these cases, must have been exceedingly active, as, anterior to the era when the Arabians cultivated medicine, the cathartics in use were exclusively of the most drastic kind. Nevertheless, we do not perceive in any one work which has descended to us, any of those dangerous consequences pointed out or alluded to, that have so much alarmed modern practitioners, and deterred them from the employment of purgatives.

Great, however, as was the homage paid to the opinion of Sydenham, it did not prevent occasional dissents from it. In turning over some of the treatises on this disease, in reference to the present inquiry, I was surprised to find how many writers there are, who, contemning his authority, had adopted the ancient practice. Thus Cheyne acquaints us, that in his time, "some eminent physicians had so little regard for the opinion of Sydenham in this matter, that, in the fit of the gout itself, they never scrupled to *drive it off*, both in themselves and others, by *strong*, *quick*, *and active purges*."

Even Sydenham himself, admits, notwithstanding all his theoretical prejudices, that, during the operation of purgatives, the patient feels no pain, or at least very little, and that if the cathartics were kept up for several days, he would most probably be cured.

It is, moreover, an interesting circumstance, and one which strikingly illustrates the propriety of the course for which I am contending, that almost all the remedies of any reputation, whether they be the preparations of regular bred physicians, or of empirics, act powerfully on the bowels, and contain for the most part, as a basis, the hermodactylus or extract of scammony.

It appears, especially, that during the prosecution of the experiments which were so abundantly made in Europe, with different substances, for the purpose of detecting the leading ingredients of the *Eau Medicinale*, an article of which I am subsequently to speak, by a resemblance in effects, that several of the more drastic cathartics, as the Elaterium and white hellebore, proved of service in gout. These, confessedly among the most harsh and violent of all our purgatives, are represented as having evinced such considerable powers in certain cases, as very generally to be considered at the time, as the chief constituents in the French nostrum. It further appears, that formerly, the Gamboge, a purge most violently distressing to the bowels, had acquired so much repute, that on this account, the title of "gutta ad podagram," was conferred upon it.

(*To be continued.*)

